# Exercise-induced changes of CSF vascular endothelial growth factor in adult chronic hydrocephalus patients

**DOI:** 10.1186/2045-8118-12-S1-O58

**Published:** 2015-09-18

**Authors:** Mark Gregory Luciano, Jun Yang, Kaitlyn J Shanahan, Leah P Shriver

**Affiliations:** 1 Cleveland Clinic, USA; 2 University of Akron, Depts. Chemistry and Biology, USA

## Background

Vascular endothelial growth factor (VEGF) is a growth factor demonstrated to play a key role in cerebral angiogenesis and neurogenesis. It has been considered a critical component in hippocampus neurogenesis and memory formation and has been observed to increase in the rat hippocampus after exercise. In a previous study, we found increases in VEGF receptor and/or ligand in an experimental model of chronic e hydrocephalus in several brain areas and cerebrospinal fluid (CSF), suggesting a role in the adaption to chronic hypoxia. Here we investigate the ability of moderate exercise to increase CSF-VEGF levels in adult chronic hydrocephalus patients.

## Methods

Lumbar CSF samples were collected from 17 normal pressure hydrocephalus (NPH) patients over 5 hours in 1-h intervals. During CSF collection, 11 patients (exercise group) underwent a standard in-room physical therapy session; 6 patients (no-exercise group) did not undergo a physical therapy session. CSF-VEGF levels were evaluated for increase related to exercise and the clinical response to CSF drainage.

## Results

CSF-VEGF levels in the exercise group demonstrated significant increases 1-3hrs post-exercise compared with the levels 1-2hrs pre-exercise (p=0.04), and also showed significantly higher levels than the no-exercise groups (p=0.03). While patients who clinically improved with CSF removal did not demonstrate an increase in CSF-VEGF levels, those who did not clinically improve had higher CSF-VEGF levels after exercise. The post-exercise CSF-VEGF level in the group that did not clinically improvement was significantly higher than both their own pre-exercise level (p=0.02) and also higher than that seen in the clinically improving group (p=0.05) after exercise.

## Conclusions

CSF-VEGF levels can increase after moderate exercise even in elderly hydrocephalus patients. This suggests a potential benefit of exercise in benefiting some of these patients may exist via a central VEGF mechanism. Increased VEGF levels after exercise in patients who showed no improvement with CSF drainage suggest that vascular injury may play a role in this group's pathophysiology.

